# Academic research and training to advance global agriculture through quantitative genetics: a personal perspective on the contributions of Rohan Fernando

**DOI:** 10.1186/s12711-024-00906-6

**Published:** 2024-05-03

**Authors:** Liviu Radu Totir

**Affiliations:** Breeding Technologies, Seed Product Development, Corteva Agriscience, Johnston, IA 50131 USA

Rohan Fernando is known and celebrated for many outstanding technical contributions to Animal Breeding and Quantitative Genetics [1]. The intent of this Editorial is to provide a personal perspective on the impact of Rohan’s scientific and pedagogical excellence on global agriculture. In the animal breeding and genetics community, it is well known that Rohan has played key technical roles in multiple public/private partnerships that resulted in measurable improvements in animal agriculture. What is less known is that Rohan has also made important contributions towards the productivity and resilience of the seed industry and thus plant agriculture.

I am a former graduate and post-doctoral student of Rohan, working under his supervision from August 1995 to September 2004, first at the University of Illinois at Urbana Champaign (UIUC) and then at Iowa State University (ISU). I joined DuPont Pioneer—now Corteva Agriscience, one of the leading global Agriscience companies, in October 2004. Here, I have spent my entire career working with teams that develop and deploy methodology and software for optimized breeding analytics and decision systems to accelerate global crop improvement. Given this background, I will provide a personal perspective on Rohan’s contributions to the seed industry and thus plant agriculture.

The seed industry is a key component in building productive, resilient, and sustainable agricultural systems (Fig. 1).

**Fig. 1:**
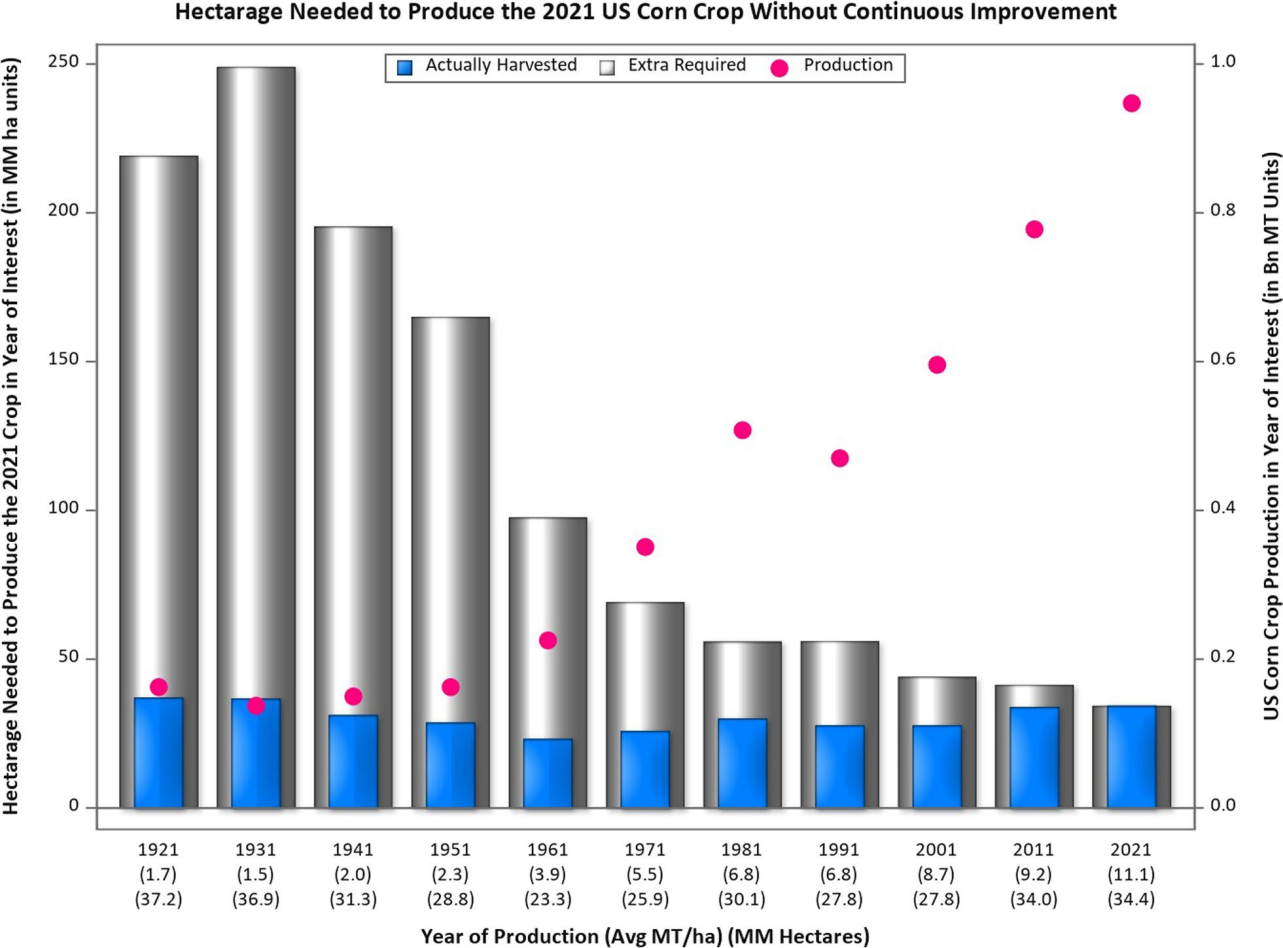
Example of the outcome of continuous improvement in US corn (maize) yield, measured in terms of land mass kept out of production (left Y axis) because of the increased production (right Y axis) due to 6.5-fold increase in yield per hectare from 1921 to 2021 (data from https://quickstats.nass.usda.gov/)

Increased yield output per unit area of land is critical given the societal constraint of restricted land use for agriculture [2]. To achieve this, modern plant agriculture makes use of scientific and technological expertise from a very wide range of domains, in an integrated and coordinated systems-based approach. The coordinated use of applied statistics, quantitative genetics, statistical computing, and decision science, focused on optimization of artificial selection within plant breeding programs, is a critical lever used to increase agricultural productivity per hectare. Given this context, the impact of Rohan’s career on the seed industry can be traced back to two main areas: (1) creation of clear scientific and software roadmaps for optimized use of novel applied statistics, quantitative genetics, and statistical computing techniques, and (2) training of graduate and post-doctoral students in a style that enabled them to seamlessly use skill sets learned in the field of animal breeding for plant breeding.

## Academic research

First, I will address the impact of the scientific and software roadmaps that were created by Rohan during his career. When looking at the trajectory of his scientific career, as reflected by his more than 200 peer-reviewed publications, three main areas of research can be identified: (1) theory and software to enable optimized data-driven artificial selection and breeding program design e.g. [3–7]; (2) theory and software for integration of genomic data in breeding—from marker assisted best linear unbiased prediction (MABLUP) to genomic selection e.g. [8–12]; and (3) theory and software for genotype probability computations in pedigreed populations under various inheritance models e.g. [13–16]. All three research areas are critical for genetic improvement in both animal and plant agriculture. As illustrated by the selected references, Rohan built roadmaps in these research areas by (1) leveraging a unique and exquisite knowledge and understanding of applied and theoretical statistics, quantitative genetics, and high-performance scientific computer programming and (2) always fostering inclusive and collaborative work patterns with students and collaborators. While working with Rohan, I noticed that he always used a 3-step “systems” approach for any new algorithm, idea, theory, or programming language to be added into his scientific roadmaps. First, in-depth discussions focused on strengths and weaknesses, second doubling down on understanding all relevant technical nuances, and third programming it from scratch within cohesive high performance software platforms. Because of this 3-step “systems” approach to research, in addition to his many peer-reviewed publications, Rohan co-authored several high-performance scientific programs with students and collaborators such as MATVEC [17] and GenSel [18] both programmed in C +  + , and JWAS [19] programmed in Julia. Each of these highly complex software platforms could have become the crowning achievement of many scientific careers. However, Rohan viewed them only as steppingstones towards achieving a complete understanding of the scientific problem at hand, training his students, and helping advance scientific knowledge relevant to agriculture. As a result, in addition to the roadmap type publications that brought clarity and understanding of critical technical aspects of the research areas he worked on (e.g., his large number of publications on whole-genome analysis and genomic selection, starting in 2006), the software packages co-authored by Rohan enabled the community to apply these learnings in large scale practical use cases [20, 21].

Rohan’s scientific publication record contains many contributions to plant breeding e.g. [22, 23] with the most recent one in 2023 [24]. The seed industry has been one of the beneficiaries of Rohan’s 3 step “systems” approach to solving complex but practical genetic evaluation problems. For example, as part of a DuPont Pioneer—ISU collaboration, I had the opportunity to work one day a week with Rohan at ISU from 2005 through 2011. These weekly one on one working sessions focused primarily on discussing and understanding the intricacies of the methodology and software development strategies relevant to the emerging whole-genome analysis and genomic selection domains. Rohan was always passionate about making sure that these emerging domains would be more than just a theoretical exercise, and that they should be implemented by plant breeders to aid in the efficient creation of new and improved crop varieties for the global grower community. Thus, our collaborative efforts helped DuPont Pioneer/Corteva Agriscience bring better seed products to our global customers faster. This helped increase the productivity per unit area of land. Thomas Jefferson famously said that “The greatest service which can be rendered any country is to add a useful plant to its culture” [25]. Through the collaboration with DuPont Pioneer/Corteva Agriscience, Rohan was able to provide this great service not just to one country, but to humankind.

## Training

Next, I will address the way Rohan trained graduate and post-doctoral students. When I look back at the many years I spent with him, first as a student and then as a collaborator, I recognize once more a “systems” approach to training. From my perspective, Rohan trained his students by focusing on four behaviors: (1) kindness, (2) technical excellence, (3) unselfish collaboration, and (4) respectful inquisitiveness. In the following, I will use my personal experience to discuss some aspects of this training approach.

I first met Rohan in August 1995 when I arrived at UIUC from Romania to start my graduate studies in Animal Breeding and Genetics. Knowing that I did not have a place to stay, Rohan offered me temporary accommodation in his home although Margie Fernando (Rohan’s third child) was born the week prior to my arrival. This was the first of many instances when Rohan showed kindness towards me without asking anything in return. This not only made me feel valued, but also realize that kindness is a critical lever in building cohesive technical teams. While always kind, Rohan made it very clear from the beginning that achieving technical excellence in applied and theoretical statistics, quantitative genetics, and scientific programming is a requirement to be successful in his program. Without realizing it, by striving to achieve technical excellence in Rohan’s program, I was also building the foundation that allowed me to seamlessly transition from animal to plant breeding. The remarkable fact was that, to help me achieve the level of technical excellence expected, Rohan spent countless hours working side by side with me on algorithms and theory, programing in C +  + , and writing drafts of scientific articles. Through this, I learned that kindness and technical excellence go hand in hand and are very effective when used together. The importance of unselfish collaboration was the next lesson I learned from Rohan. During my tenure with Rohan, he approached all scientific engagements in a very unselfish collaborative manner. His primary focus was always on solving the scientific problem of interest without worrying about getting credit for his contributions. This unselfish collaborative approach, combined with his outstanding technical excellence and kindness, made Rohan a highly sought-after and respected collaborator for many distinguished scientists such as Charles Henderson, Dan Gianola, Daniel Sorensen, Moshe Soller, Bill Hill, Robert Elston, Jack Dekkers, Dorian Garrick, Albrecht Melchinger etc. many of whom he wrote publications with. However, Rohan treated students and early career scientists with the same unselfish collaborative spirit, thus helping many of them move forward on their projects through mentorship and guidance. Finally, Rohan always role modeled to his students the need for respectful inquisitiveness when working on problems where the expertise resides in other fields of science. I recall us spending many hours in the late 1990’s early 2000’s discussing fine points and intricacies of Markov chain Monte Carlo (MCMC) theory with Wolfgang Kliemann who was a Mathematics professor at ISU or discussing graph theory and high-performance parallel computing programming with various faculty in Computer Science. I also recall taking, at Rohan’s recommendation, a class in Measure Theory in the Mathematics Department at ISU to try to better understand the foundational theory behind MCMC. While at the time I did not see the immediate practical value of these scientific domains, which were also rather difficult for me to understand, it became clear in time that Rohan was in fact setting the foundation needed for him to become one of if not the foremost technical expert in Bayesian MCMC methodology and computational software applied to agriculture. Through his actions, he was teaching his students the value of respectful inquisitiveness when working on complex problems that require interactions with experts from other scientific domains.

In my experience, all four behaviors cultivated by Rohan in his training system are essential to succeed as a technical expert in the seed industry and more broadly plant agriculture. This is especially true in a global research and development organization, like the one at Corteva Agriscience, that operates based on multi-cultural teamwork, collaboration, agility and continuous integration of new science and technology into large-scale production systems.

## Conclusions

From a personal perspective, it has become second nature for me to leverage Rohan’s “systems” based approach to research and training as we develop new scientific roadmaps at Corteva Agriscience. A relevant example is the scientific roadmap that we have developed for the integration of crop growth models with whole genome prediction [26, 27]. I argue that we have developed this new roadmap by leveraging most if not all the components role modeled by Rohan throughout his career: (1) form a cohesive team with a talented early career post-doctoral scientist (Frank Technow 2014 to 2015); (2) establish unselfish collaboration and respectful inquisitiveness based working patterns with crop growth modeling experts (Charlie Messina and Mark Cooper); and (3) adopt and promote the 3-step “systems” approach to enable additional collaborators to help evaluate and explore the value of the new roadmap developed [28]. This editorial provides a personal perspective on the impact of Rohan Fernando on the seed industry and thus plant agriculture. However, it is likely an incomplete view given that several other former students of Rohan work in key roles in the seed industry for companies such as: BASF, Bayer, and Corteva Agriscience. It is my hope that I have managed to bring to light some of the contributions made by Rohan to global agriculture, and maybe encourage others to complete the picture with their perspectives.

## Data Availability

not applicable.
